# Improvement of l-Leucine Production in *Corynebacterium glutamicum* by Altering the Redox Flux

**DOI:** 10.3390/ijms20082020

**Published:** 2019-04-24

**Authors:** Ying-Yu Wang, Feng Zhang, Jian-Zhong Xu, Wei-Guo Zhang, Xiu-Lai Chen, Li-Ming Liu

**Affiliations:** 1 The Key Laboratory of Industrial Biotechnology, Ministry of Education, School of Biotechnology, Jiangnan University, 1800# Lihu Road, Wuxi 214122, China; wangyy0711@163.com (Y.-Y.W.); zhang20180606@sina.com (F.Z.); 2 The Key Laboratory of Carbohydrate Chemistry and Biotechnology, Ministry of Education, School of Biotechnology, Jiangnan University, 1800# Lihu Road, Wuxi 214122, China; 3 State Key Laboratory of Food Science and Technology, Jiangnan University, Wuxi 214122, China; shirleyl1205@163.com (X.-L.C.); peidongchen13@163.com (L.-M.L.)

**Keywords:** *Corynebacterium glutamicum*, l-leucine, acetohydroxyacid isomeroreductase, leucine dehydrogenase, NAD-dependent glutamate dehydrogenase

## Abstract

The production of l-leucine was improved by the disruption of *ltbR* encoding transcriptional regulator and overexpression of the key genes (*leuAilvBNCE*) of the l-leucine biosynthesis pathway in *Corynebacterium glutamicum* XQ-9. In order to improve l-leucine production, we rationally engineered *C. glutamicum* to enhance l-leucine production, by improving the redox flux. On the basis of this, we manipulated the redox state of the cells by mutating the coenzyme-binding domains of acetohydroxyacid isomeroreductase encoded by *ilvC*, inserting NAD-specific leucine dehydrogenase, encoded by *leuDH* from *Lysinibacillus sphaericus*, and glutamate dehydrogenase encoded by *rocG* from *Bacillus subtilis*, instead of endogenous branched-chain amino acid transaminase and glutamate dehydrogenase, respectively. The yield of l-leucine reached 22.62 ± 0.17 g·L^−1^ by strain ΔLtbR-acetohydroxyacid isomeroreductase (AHAIR)^M^/ABNC^M^E, and the concentrations of the by-products (l-valine and l-alanine) increased, compared to the strain ΔLtbR/ABNCE. Strain ΔLtbR-AHAIR^M^LeuDH/ABNC^M^LDH accumulated 22.87±0.31 g·L^−1^ l-leucine, but showed a drastically low l-valine accumulation (from 8.06 ± 0.35 g·L^−1^ to 2.72 ± 0.11 g·L^−1^), in comparison to strain ΔLtbR-AHAIR^M^/ABNC^M^E, which indicated that LeuDH has much specificity for l-leucine synthesis but not for l-valine synthesis. Subsequently, the resultant strain ΔLtbR-AHAIR^M^LeuDHRocG/ABNC^M^LDH accumulated 23.31 ± 0.24 g·L^−1^ l-leucine with a glucose conversion efficiency of 0.191 g·g^−1^.

## 1. Introduction

l-leucine, one of three branched-chain amino acids (BCAAs), is an essential amino acid that is not synthesized in mammals, and is used in food and animal feed additives, pharmaceuticals, and cosmetics [1,2,3,4]. l-leucine acts as potent nutritional signaling molecules for regulating the rate of protein synthesis and glucose homeostasis [2,5]. Therefore, l-leucine has also been widely used as additives of infusion solutions, together with the other BCAAs for patients with hepatic diseases, to improve the nutritional status [2].

**Figure 1:**
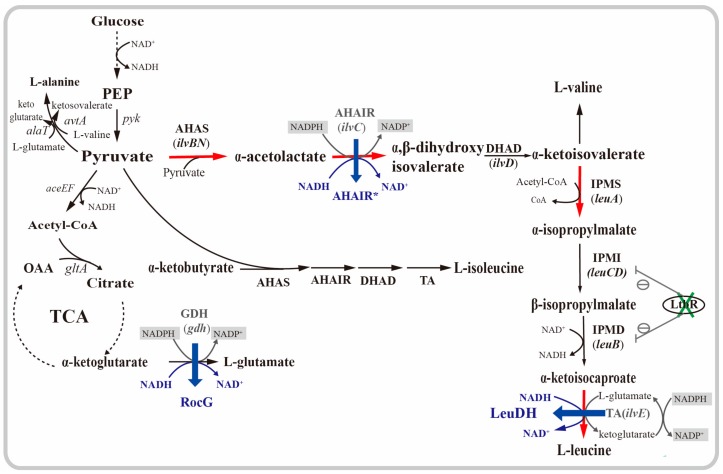
The l-leucine biosynthesis pathway of *Corynebacterium glutamicum* and the metabolic engineering steps performed in this study. Enzymes and corresponding genes are shown. An encircled minus indicates repression of gene expression (solid lines). Deletion of the gene encoding LtbR is indicated by green “×”. Red thick arrows indicate increased metabolic fluxes. Introduction of enzymatic reactions are highlighted in blue. NADPH and NADP^+^ are highlighted in grey shaded boxes. Abbreviations: AHAS—acetotydroxyacid synthetase; AHAIR—acetohydroxyacid isomeroreductase; DHAD—dihydroxyacid dehydratase; TA—branched-chain amino acid transaminase; IPMS—α-isopropylmalate synthase; IPMI—α-isopropylmalate isomerase; IPMD—β-isopropylmalate dehydrogenase; LeuDH—leucine dehydrogenase; PEP—phosphoenolpyruvate; OAA—oxaloacetic acid; TCA—tricarboxylic acid cycle; GDH and RocG—glutamate dehydrogenase.

*Corynebacterium glutamicum* is widely used in industrial fermentation to produce several million tons of amino acids annually, in particular the flavor enhancer l-glutamate and the feed additive l-lysine [6,7]. Besides l-glutamate and l-lysine, *C. glutamicum* can also be used for production of a variety of other amino acids, including the BCAAs [8,9,10]. To date, *C. glutamicum* mutant strains remain the dominant industrial producers of l-leucine [11]. At present, microbial fermentation is the major method for producing l-leucine because of the advantages of suitable reaction conditions, environmental friendliness, and a stable product quality [12]. Nowadays, the global production capacity of l-leucine is about 1000 tons per year, though the market is substantially growing [13]. There is a need for developing more effective production methods, such as giving high yields, to meet growing market demands [14,15].

l-leucine is synthesized from pyruvate in a pathway comprising seven reactions (Figure 1), catalyzed by acetohydroxyacid synthase (AHAS), acetohydroxyacid isomeroreductase (AHAIR), dihydroxyacid dehydratase (DHAD), α-isopropylmalate synthase (IPMS), α-isopropylmalate isomerase, β-isopropylmalate dehydrogenase (IPMD), and branched-chain amino acid transaminase (TA) [16]. The l-leucine biosynthetic pathway is complex and tightly regulated because the overlapping biosynthesis pathways of the three BCAAs partly share the same precursors and enzymes [8]. l-valine and l-isoleucine are synthesized in parallel pathways in which the reaction steps are catalyzed by the same enzymes, whereas l-leucine is formed in a specific series of four reactions, starting with the last intermediate of l-valine biosynthesis [17]. Two genes, *alaT* or *avtA*, are responsible for the conversion of pyruvate to l-alanine. Additionally, the gene *leuB* together with the genes *leuCD* is repressed by the transcriptional regulator LtbR. Therefore, the regular strategies for the construction of l-leucine producing strains are, removing the l-leucine inhibition and transcriptional attenuation (*leuA*, *ilvBNC*) [3,18], enhancing the metabolic influx (*leuACDB*, *ilvBNCE*) to precursor [8], altering substrate and enzyme specificity [19], and so on. However, improvement of the redox cofactor requirement for the l-leucine production has not been investigated.

As shown in Figure 1, there is a net consumption of reducing equivalents from NADPH, during the l-leucine biosynthesis. The synthesis of one mole of l-leucine consumes a total of two moles of NADPH. The consumption of NADPH is at the AHAIR reaction and at the regeneration of l-glutamate as an amino-group donor for the TA reaction [6]. The importance of NADPH supply for amino acid production is well-known [20,21]. A limited supply of reducing equivalents might be a suspected rate-limiting factor in l-leucine production by *C. glutamicum* [7]. In this study, we report the rational design of l-leucine producer *C. glutamicum* XQ-9, with a double l-isoleucine and l-methionine auxotrophy. Recombinant strains were constructed by deleting *ltbR* and the overexpressing *leuAilvBNCE*. Then, we improved the l-leucine production in *C. glutamicum* strains by improving the redox balance. These strategies were via the modification of the coenzyme specificity of AHAIR, introduction of NAD-specific glutamate dehydrogenase (RocG) (GenBank: NC_000964.3), derived from *Bacillus subtilisI* and NAD-specific leucine dehydrogenase (LeuDH) (GenBank: AB103119.1) derived from *Lysinibacillus sphaerius*, instead of glutamate dehydrogenase (GDH) and TA [22,23,24]. All mutant AHAIR, RocG, and LeuDH consumed NADH in their reactions, and regulated cofactor production and consumption, during l-leucine biosynthesis (Figure 1). As a result, the l-leucine production and NADPH/NADP^+^ ratio were significantly increased.

## 2. Results

### 2.1. Effect of Deletion of ltbR and Expression of Genes Involved in Leucine Biosynthesis

**Figure 2:**
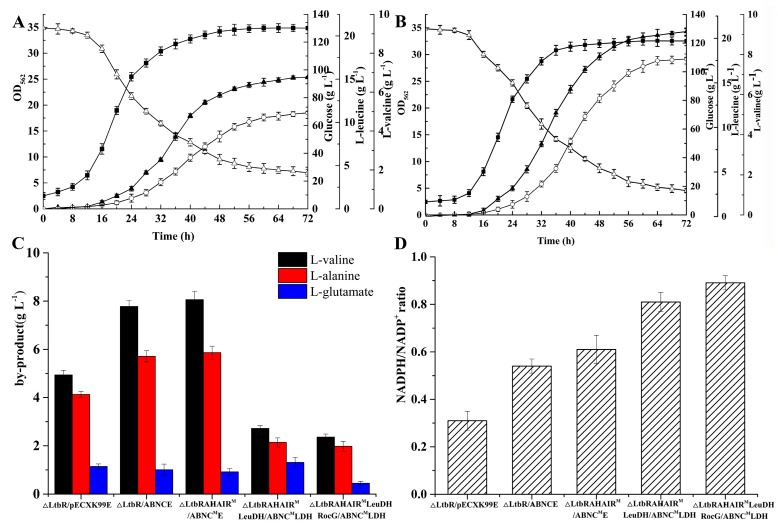
Comparison of the different *C. glutamicum* strains during cultivation in shake-flasks with the fermentation medium, to test the effect of inserting *leuAilvBNCE*. (**A**) ΔLtbR /pECXK99E (pECXK99E was used here as a control plasmid), (**B**) ΔLtbR/ABNCE, (**C**) by-products, and (**D**) NADPH/NADP^+^ ratio. Solid squares—OD_562_, hollow triangles—glucose, solid triangles—l-leucine: hollow circles—l-valine. The data represent the mean values and standard deviations obtained from three independent cultivations.

The strain *C. glutamicum* XQ-9 is auxotrophic for l-isoleucine and l-methionine (see Section 4). It can produce 14.12 g·L^−1^ l-leucine, and the productivity was 0.196 g·L^−1^·h^−1^. We sequenced the key genes in *C. glutamicum* XQ-9, and sequences of the key genes can be found in the supplementary materials. There are many amino acid exchanges in *C. glutamicum* XQ-9 with respect to the corresponding sequence from the wild-type strain ATCC 13032, some of which must be potentially responsible for the fact that *C. glutamicum* XQ-9 accumulated 14.12 g·L^−1^ l-leucine, while ATCC 13032 typically did not [3]. Especially, there were six amino acid exchanges (G92D, I162V, N210D, E216K, R494H, and G526D) encoded by the *leuA* gene. Since the transcriptional regulator LtbR was shown to repress the expression of *leuCD* and *leuB* [3], *ltbR* was deleted in *C. glutamicum* XQ-9. In order to increase the metabolic carbon flux and improve the redox balance, the *leuAilvBNCE* genes from *C. glutamicum* XQ-9 were cloned into the expression vector pEC-XK99E, resulting in plasmid pEC-ABNCE. The plasmid was transferred into strain ΔLtbR, and the enzyme activities of crude extracts were determined. The *leuCD* gene encoded isopropylmalate isomerase (IPMI). In strain ΔLtbR, an IPMI activity of 167 ± 13 mU·mg_protein_^−1^ was measured, which corresponded to a 4.5-fold increase in comparison to the *C. glutamicum* XQ-9 (37 ± 9 mU·mg_protein_^−1^) (Table 1). As shown in Table 1, all enzyme activities intended to be overexpressed in ΔLtbR/ABNCE were indeed increased to a certain extent, and the intracellular NADPH/NADP^+^ ratio of ΔLtbR/ABNCE reached 0.54 ± 0.03 (Figure 2D). Both, the increased glucose consumption and the increased NADPH/NADP^+^ ratio resulted in an increased leucine biosynthesis, based on reducing equivalents from NADH.

Strain ΔLtbR /pECXK99E, as a control group, only accumulated 15.22 g·L^−1^ l-Leucine (Figure 2A). Nevertheless, l-leucine production and glucose conversion efficiency reached 20.75 g·L^−1^ and 0.183 g·g^−1^ by ΔLtbR/ABNCE (Figure 2B). However, it cannot be ignored that the concentration of l-valine was 7.78 ± 0.25 g·L^−1^ by ΔLtbR/ABNCE, i.e., higher than 4.94 ± 0.19 g·L^−1^ by ΔLtbR/pECXK99E (Figure 2C). We see this as a consequence of shared intermediates and the enzymes in the l-leucine and l-valine biosynthesis pathway [25]. The l-valine production was yet increased by the overexpressing l-leucine biosynthesis genes. Additionally, the concentration of l-alanine as the other main by-product was increased by 27.67% (from 4.13 ± 0.13 g·L^−1^ to 5.71 ± 0.23 g·L^−1^) (Figure 2C), suggesting that l-alanine synthesis might have accumulated through the reaction catalyzed by *avtA* (valine-pyruvate aminotransferases) when l-valine biosynthesis was enhanced [26]. However, the concentrations of l-glutamate did not significantly change (Figure 2D).

### 2.2. Effect of ilvC^M^ on L-Leucine Production

On the basis of the Rossmann fold in the coenzyme-binding domains, NAD-specific enzymes commonly possess the β-α-β motif and exhibit a highly conserved GXGXXGXXXG sequence (where X is any amino acid) [27,28,29]. Negatively charged glutamate or aspartate residues form hydrogen bonds to the 2′-and/or 3′-hydroxyl groups of NAD, while positively charged arginine residue corresponds to the negatively charged 2′-phosphate group of NADP [24]. AHAIR reaction requires NADPH as a coenzyme [30]. In order to further improve the formation of l-leucine, mutagenesis of AHAIR was carried out in the strain ΔLtbR, to improve the redox flux on l-leucine production. Thus, the mutation S34G corresponds to the fourth glycine residue on the NAD-specific conserved sequence, and the mutation L48E and R49F causes better association with the NAD. Accordingly, based on these observations, the mutant AHAIR with substitution of three amino acid residues (S34G, L48E, and R49F) by site-directed mutagenesis, can mainly utilize NADH as coenzyme [23]. Firstly, wild-type AHAIR and mutant AHAIR were purified for kinetic analysis from crude extracts of strains BL21/pET28a-*ilvC* and BL21/pET28a-*ilvC*^M^. Table 2 shows kinetic constants determined for α-acetolactate and coenzyme of the wild-type AHAIR and mutant AHAIR. Wild-type AHAIR had a higher affinity for NADPH, because of the 27-fold lower *Km* for NADPH than NADH (Table 2). However, *kcat/Km* of mutant AHAIR with NADPH, decreased significantly, and *kcat/Km* of mutant AHAIR using NADH, was six-fold higher than that using NADPH (Table 2). These results suggested that the coenzyme specificity of mutant AHAIR was reversed from NADPH to NADH, even though mutant AHAIR activity using NADH as a coenzyme was not significantly improved.

**Figure 3:**
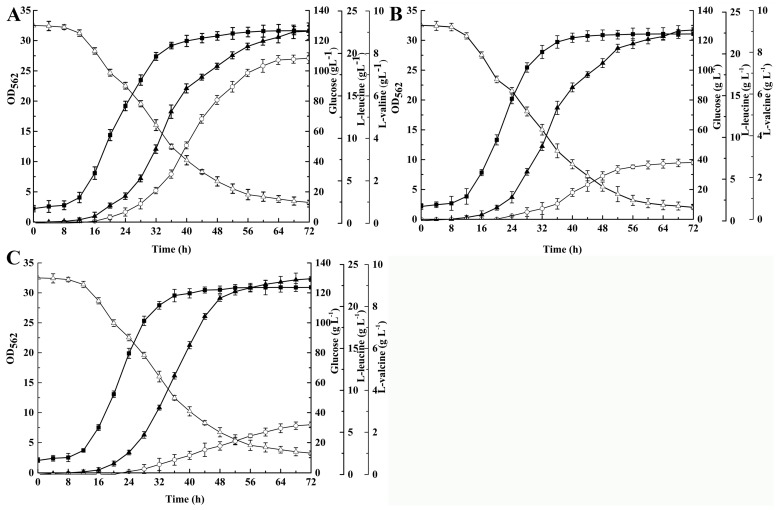
Comparison of the different *C. glutamicum* strains during cultivation in shake-flasks with fermentation medium to test the effect of additional reducing equivalents delivered by NADH. (**A**) ΔLtbR-AHAIR^M^/ABNC^M^E, (**B**) ΔLtbR-AHAIR^M^LeuDH*/*ABNC^M^LDH, and (**C**) ΔLtbR-AHAIR^M^LeuDHRocG*/*ABNC^M^LDH. Solid squares—OD_562_, hollow triangles—glucose, solid triangles—l-leucine, hollow circles—l-valine. The data represent the mean values and standard deviations obtained from three independent cultivation.

Then, mutagenesis was carried out in strain *C. glutamicum* ΔLtbR with pK18*mobsacB*-*ilvC*^M^ via two-step homologous recombination, and the correct integration was verified by sequencing. With the *leuAilvBNC*^M^*E* genes over-expressed, the l-leucine production and conversion efficiency were enhanced to 22.62 ± 0.17 g·L^−1^ and 0.193 g·g^−1^ by ΔLtbR-AHAIR^M^/ABNC^M^E, compared with ΔLtbR/ABNCE (Figure 2B and Figure 3A). The productivity reached 0.314 g·L^−1^.h^−1^ by ΔLtbR-AHAIR^M^/ABNC^M^E. Meanwhile, the intracellular NADPH/NADP^+^ ratio of ΔLtbR-AHAIR^M^/ABNC^M^E was 0.61 ± 0.06, a little higher than that of ΔLtbR/ABNCE (Figure 2D). Additionally, strain ΔLtbR-AHAIR^M^/ABNC^M^E with the mutated *ilvC* showed a notably slower growth (Figure 3A), compared to ΔLtbR-AHAIR^M^/ABNC^M^E (Figure 2B and Figure 3A). Already this could account for the 9% increase in the l-leucine formation. The concentrations of the two byproducts (8.06 ± 0.35 g·L^−1^ l-valine and 5.86 ± 0.27 g·L^−1^ l-alanine) were not significantly increased and were very much similar to the byproduct formation of ΔLtbR/ABNCE, while l-leucine production was enhanced in ΔLtbR-AHAIR^M^/ABNC^M^E (Figure 2C and Figure 3A). Consequently, ΔLtbR-AHAIR^M^ was used for further studies.

### 2.3. Effect of leuDH to Reduce l-Valine Production

Leucine dehydrogenase LeuDH, encoded by *leuDH*, is a NAD(H)-dependent amino acid dehydrogenase, catalyzing the reductive amination of a variety of aliphatic keto-acids to the corresponding l-amino acids [31]. The reaction is reversible using NAD^+^ as a factor (Figure 1). LeuDH was mainly found in several *Bacillus* strains, such as *Bacillus cereus*, *B. stearothermophilus*, *Lysinibacillus sphaericus* [32,33,34]. However, this NAD-specific amino acid dehydrogenase did not occur in *C. glutamicum*. Hence, in order to introduce LeuDH and abolish the aminotransferase reaction, we first deleted *ilvE* in ΔLtbR-AHAIR^M^, to generate the leucine-auxotrophic strain ΔLtbR-AHAIR^M^ΔTA. As expected, the growth of this strain was severely restricted, and the consumption of glucose was at a low level because the pathway of glucose to BCAAs was blocked. The similar results were reported by LiYan Feng [19]. Subsequently, this strain was transformed with pk18*mobsacB*-Δ*ilvE::leuDH*, carrying the *leuDH* gene from *L. sphaericus*, under the control of the P_tac_ promoter, and the correct integration was verified by sequencing.

With the *leuAilvBNC^M^leuDH* genes being over-expressed, the resulting strain, ΔLtbR-AHAIR^M^LeuDH/ABNC^M^LDH, accumulated approximately 22.87 ± 0.31 g·L^−1^ l-leucine, and the intracellular NADPH/NADP^+^ ratio was slightly increased to 0.81 ± 0.04 (Figure 2C and Figure 3B). Additionally, glucose conversion efficiency and productivity reached 0.187 g·g^−1^ and 0.318 g·L^−1^·h^−1^, respectively. Surprisingly, the concentrations of the by-products l-valine (2.72 ± 0.11 g·L^−1^) and l-alanine (2.14 ± 0.19 g·L^−1^) were at a lower level. Perhaps branched-chain aminotransferase mainly catalyzed the BCAAs biosynthesis and LeuDH had a higher preference for l-leucine biosynthesis than for l-valine biosynthesis. In addition, l-glutamate production reached 1.31 ± 0.21 g·L^−1^, which was higher than other *C. glutamicum* strains. l-glutamate served as the amino group donor for several amino acids, except in the ΔLtbR-AHAIR^M^LeuDH strains [35]. This is because LeuDH uses NH_3_ as the amino-group donor and NADH as a cofactor [32]. In *C. glutamicum* strains, to produce l-leucine, adding additional l-glutamate in the fermentation medium might increase the l-leucine production. This result indicated that the fermentation progress of ΔLtbR-AHAIR^M^LeuDH/ABNC^M^LDH did not require any additional l-glutamate supply. Although the l-leucine production was lower than expected, it had a significant positive influence on product composition. Strain ΔLtbR-AHAIR^M^LeuDH was used for further improvement of the l-leucine production.

### 2.4. Effect of rocG in L-Leucine Production

*B. subtilis* glutamate dehydrogenase RocG, encoded by *rocG*, converts the formation of glutamate, through the reductive amination of 2-ketoglutarate [36]. The reaction requires NADH as a cofactor [22]. The expression of *rocG* to replace endogenous glutamate dehydrogenase might improve the redox balance for l-leucine biosynthesis [22]. Hence, in order to introduce *rocG* and abolish the endogenous glutamate dehydrogenase reaction, ΔLtbR-AHAIR^M^LeuDHRocG was constructed with pk18*mobsacB*-Δ*gdh::rocG*, carrying the *rocG* gene under the control of the P_tac_ promoter, and the correct integration was verified by sequencing. As shown in Table 1 and Figure 3C, with the *leuAilvBNC^M^leuDH* genes over-expressed, RocG activity of 107 ± 13 mU·mg_protein_^−1^ was measured, and the concentration of l-leucine was increased to 23.31 ± 0.24 g·L^−1^, with a glucose conversion efficiency of 0.191 g·g^−1^. The productivity reached 0.324 g·L^−1^·h^−1^. Additionally, the intracellular NADPH/NADP^+^ ratio reached 0.89 ± 0.03, possibly reducing more equivalents from NADH for the l-leucine accumulation. However, the l-glutamate production dramatically decreased to 0.45 ± 0.08 g·L^−1^ by ΔLtbR-AHAIR^M^LeuDHRocG/ABNC^M^LDH. Perhaps the RocG catalyzed reaction was reversible, and l-glutamate was degraded by RocG in ΔLtbR-AHAIR^M^LeuDHRocG/ABNC^M^LDH [37]. Under this condition, glucose consumption and cell growth showed no dramatic difference compared to ΔLtbR-AHAIR^M^LeuDH/ABNC^M^LDH (Figure 3B,C).

## 3. Discussion

NADPH plays a crucial role in the biochemical reactions, and has several physiological functions in the amino acid-producing strains, acting as a key cofactor in metabolism, especially anabolism. As shown in Figure 1, there is a net consumption of the reducing equivalents from NADPH, during the l-leucine biosynthesis. In order to make use of the additional reducing equivalents delivered by NADH, the coenzyme specificity of AHAIR was reversed, and NAD-specific LeuDH and RocG were introduced, instead of the endogenous TA and GDH, respectively.

While *kcat/Km* of the mutant AHAIR using NADH was slightly lower than that of the wild-type AHAIR (Table 1), the mutant AHAIR preferred NADH as a cofactor over NADPH. The intracellular NADPH concentration in ΔLtbR-AHAIR^M^/ABNC^M^E was enhanced in comparison to ΔLtbR/ABNCE, while the AHAIR activity in ΔLtbR-AHAIR^M^/ABNC^M^E was decreased (in the supplementary materials). The intracellular NADPH concentration determined during l-leucine production reached 3.92 ± 0.33 μmol g^−1^, compared to 3.48 ± 0.21 μmol g^−1^ of △LtbR/ABNCE (in the supplementary materials). Thus, it appears that the mutant AHAIR (S34G, L48E, and R49F) might indeed redirect redox fluxes and be able to work efficiently for L-leucine production, using NADH [24,27,28,29].

LeuDH, which is an amino acid dehydrogenase using NADH as a cofactor, catalyzes the reversible oxidative deamination and reductive amination between l-leucine or other branched chain amino acids and their corresponding α-keto acids [38]. Although various active or artificial amino acids have been synthesized by leucine dehydrogenases in vitro [6,31], the effect of leucine dehydrogenases in biological l-leucine fermentation has not yet been recognized in detail. Nevertheless, the NAD-dependent LeuDH was expected to be effective for l-leucine biosynthesis. In fact, LeuDH improved intracellular redox balance and had much more specificity for the biosynthesis of l-leucine than for the biosynthesis of l-valine [26]. It could be seen that the concentration of the by-product l-valine (2.72 ± 0.11 g·L^−1^) was at a low level for ΔLtbR-AHAIR^M^LeuDH/ABNC^M^LDH. As expected, the NAD-preferring LeuDH significantly increased the NADPH/NADP^+^ ratio. As known, LeuDH uses NH_3_ as an amino-group donor, whereas TA has a major preference for l-glutamate, and NADPH is consumed for the regeneration of l-glutamate [39]. Thus, the demand of an amino-group donor for l-leucine synthesis was changed from l-glutamate to NH_3_ in *C. glutamicum* XQ-9Δ*ltbRilvC*^M^Δ*ilvE::leuDH* strains. Indeed the demand of l-glutamate in ΔLtbR-AHAIR^M^LeuDH strains was decreased, and l-glutamate was accumulated (Figure 1 and Figure 2C). On the other hand, in order to ensure sufficient amounts of NH_3_ for LeuDH, adding NH_3_ to maintain the pH of the fermentation solution could be easily provided.

In order to further improve the balance of the redox fluxes, the NAD-dependent glutamate dehydrogenase *rocG* was used for the l-leucine production. As a result, ΔLtbR-AHAIR^M^LeuDHRocG/ABNC^M^LDH produced 23.31±0.24 g·L^−1^ l-leucine. Additionally, the NADPH/NADP^+^ ratio reached 0.89 ± 0.03, slightly higher than 0.81 ± 0.04 by ΔLtbR-AHAIR^M^LeuDH/ABNC^M^LDH. However, l-glutamate only accumulated to a concentration of 0.45 ± 0.08 g·L^−1^ by ΔLtbR-AHAIR^M^LeuDHRocG/ABNC^M^LDH. Depending on the cofactor and metabolite levels, *B. subtilis* RocG converts ketoglutarate to l-glutamate, in the presence of NADH or catalyzes the reverse reaction. RocG can catalyze the reductive deamination of glutamate to form ketoglutarate, and thus, enable a direct utilization of glutamate as a carbon and nitrogen source [36]. We interpret the lower l-glutamate production of this strain to be a consequence of the intracellular levels of NAD^+^, NADH, ketoglutarate, and l-glutamate, so that RocG catalyzes the interconversion in the direction of l-glutamate consumption. Presumably, this process makes part of a futile cycle with l-glutamate production through the GS/GOGAT system, which has a high capacity when GDH is absent [37].

The regular strategies to improve the l-leucine yield have largely focused on the metabolic engineering of removing feedback inhibition [18,40,41], upstream central carbon flux [3], and downstream by-product synthesis pathways [8]. This is the first report on improvement in redox flux, to enhance the production of l-leucine by *C. glutamicum*. The mutant AHAIR (S34G, L48E, and R49F) not only increased the NADPH/NADP^+^ ratio but also increased the product l-leucine and the by-product production. LeuDH has much specificity for l-leucine biosynthesis and not for l-valine biosynthesis; thereby, it will significantly ease or improve product purification. RocG may well have catalyzed conversion of l-glutamate into ketoglutarate.

## 4. Materials and Methods

### 4.1. Bacterial Strains, Plasmids, and Culture Conditions

All strains and plasmids used in this study as well as their relevant characteristics are listed in Table 3. *C. glutamicum* XQ-9 was engineered by repeated random mutagenesis and through directed selection from wild type *C. glutamicum* ATCC 13032. *C. glutamicum* XQ-9 was resistant to α-thiazolealanine, α-aminobutyic acid, sulfaguanidine, and l-Leucin-hydroxyamid; was auxotrophic for l-isoleucine and l-methionine; could produce 14.12 g·L^−1^ l-leucine; and was used as the working and parent strain. All DNA oligonucleotides were synthesized by the General Biosystems Co. Ltd. (Anhui, China) and are listed in Table S2 (in the supplementary materials). Site-directed mutagenesis was performed using Mut Express^R^ II Fast Mutagenesis Kit V2 (Vazyme, Nanjing, China). Using this kit, the target plasmid amplification product was digested by *Dpn* I, and directly transformed after the recombinant cyclization, using recombinase Exnase II. The *leuDH* gene from *L. sphaericus* was synthesized by the General Biosystems Co. Ltd. (Anhui, China), with the addition of an SD sequence (GAAAGGAGATATACC) before ATG. We decided to not change any codons in *leuDH*, according to the different codon usage statistics in *L. sphaericus* and *C. glutamicum*. Promoter and terminator elements could be used from pDXW-8. Thus, *leuDH* fragment was cloned into the vector pDXW-8. The expression vector pET28a carrying His tag was for purification and kinetic analysis of AHAIR. The shuttle vectors pDXW-8 carrying an IPTG-inducible *tac* promoter and pEC-XK99E, were for the gene transfer between *E. coli* and *C. glutamicum*, and were used for gene overexpression in *C. glutamicum* [42]. The vector pk18*mobsacB* was for gene deletions and integrations via a two-step homologous recombination in *C. glutamicum* [19]. The plasmids constructed in this study can be found in the supplementary materials. For strain construction, plasmids were transformed into *C. glutamicum* by electroporation. All constructed plasmids including chromosomal deletions and integrations in engineered strains were finally verified by DNA sequencing.

For the recombinant DNA work, *E. coli* JM109 and BL21(DE3) were used and cultivated in a Luria–Bertani (LB) medium (5 g·L^−1^ yeast extract, 10 g·L^−1^ tryptone, and 10 g·L^−1^ NaCl), at 37 °C. Where appropriate, 50 mg·L^−1^ kanamycin or 0.1 mmol·L^−1^ isopropyl β-D-thiogalactoside (IPTG) were added to the medium. *C. glutamicum* and its recombinant derivatives were routinely cultivated aerobically at 30 °C in the LBG medium (LB medium supplemented with 5 g·L^−1^ glucose). LBHIS medium (5 g·L^−1^ tryptone, 5 g·L^−1^ NaCl, 2.5 g·L^−1^ yeast extract, 18.5 g·L^−1^ Brain Heart Infusion powder, and 91 g·L^−1^ sorbitol) was used for the transformation of the mutant gene into *C. glutamicum* cells. When necessary, 25 mg·L^−1^ kanamycin was added to the medium. Bacterial growth was followed by measuring the optical density at 562 nm (OD_562_).

The medium used for seed culture consisted of (per liter) 30 g glucose, 35 g corn steep liquor, 5 g (NH_4_)_2_SO_4_, 1.3 g KH_2_PO_4_·3H_2_O, 0.4 g MgSO_4_·7H_2_O, 0.01 g MnSO_4_·H_2_O, 10 g sodium citrate ·2H_2_O, 10 g yeast extract, 2 g urea, 0.4 g l-methionine, 200 μg biotin, 300 μg thiamine, and 20 g CaCO_3_. The fermentation medium contained (per liter) 130 g glucose, 25 g corn steep liquor, 15 g (NH_4_)_2_SO_4_, 15 g CH_3_COONH_4_, 1.3 g KH_2_PO_4_·3H_2_O, 0.5 g MgSO_4_·7H_2_O, 0.01 g MnSO_4_·H_2_O, 2 g sodium citrate ·2H_2_O, 2 g urea, 0.8 g l-methionine, 0.06 g l-isoleucine, 0.5 g l-glutamate, 100 μg biotin, 200 μg thiamine, and 30 g CaCO_3_. Both media were adjusted to pH 7.3 with NaOH.

### 4.2. Preparation of Crude Extracts and Enzyme Assays

Crude cell extracts were prepared for the determination of AHAS, AHAIR, IPMS, IPMD, TA, LeuDH, and GDH activity. Cells were grown in the LBG medium. Cultivated cells were harvested by centrifugation (8000× *g*, 20 min) at 4 °C, and washed twice with 50 mmol L^−1^ Tris-HCl. Then, they were treated with lysozyme (20 mg mL^−1^) at 37 °C for 1 h [43]. The resulting cell suspensions were sonicated, using an ultrasonic homogenizer in an ice water bath, for 15 min. Cell debris was removed by centrifugation (8000× *g*, 10 min) at 4 °C. Protein concentrations were determined using the Bradford Protein Quantification Kit (Sangon, Shanghai, China) with bovine serum albumin as standard. The analyses of enzyme activities and protein concentrations were done in triplicates.

AHAS activity was determined at 30 °C as the decrease of pyruvate absorbance at 333 nm (the extinction coefficient was 17.5 M^−1^cm^−1^) by using a Microplate reader (Molecular Devices, UAS). The reaction mixture contained 100 mmol L^−1^ potassium phosphate (pH 7.5), 50 mmol L^−1^ sodium pyruvate, 10 mmol L^−1^ MgCl_2_, 0.1 mmol L^−1^ thiamine pyrophosphate, 0.1 mmol L^−1^ flavin adenine dinucleotide, and crude extract [23]. One unit of AHAS activity was defined as the activity needed to form 1 μmol of α-acetolactate per min.

AHAIR activity was determined at 30 °C, by monitoring NADPH decrease at 340 nm. The reaction mixture contained 100 mmol L^−1^ potassium phosphate (pH 7.5), 10 mmol L^−1^ α-acetolactate, 5 mmol L^−1^ MgCl_2_, 0.2 mmol L^−1^ NADPH, and crude extract [23]. One unit of AHAIR activity was defined as the activity necessary to oxidize 1 μmol of NADPH per min.

IPMS activity was determined at 30 °C, by monitoring coenzyme A formation at 412 nm (the extinction coefficient was 13.6 M^−1^cm^−1^) [44]. The reaction mixture contained 50 mmol L^−1^ Tris-HCl buffer (pH 7.5) with acetyl-CoA solution (3 mmol L^−1^ in 50 mmol L^−1^ Tris-HCl pH 7.5), 5,5′-dithiobis-(2-nitrobenzoic acid) (DTNB) solution (1 mmol L^−1^ in 50 mmol L^−1^ Tris-HCl pH 7.5), 400 mmol L^−1^ potassium glutamate, deionized water, and crude extract [2,3,4]. The reaction was started by adding α-ketoisovalerate solution (40 mmol L^−1^ in 50 mmol L^−1^ Tris-HCl, pH 7.5). One unit of IPMS activity was defined as the activity needed to form 1 μmol of α-isopropylmalate per min.

IPMI activity was determined at 30 °C, by monitoring the reaction intermediate α-isopropylmaleate formation at 235 nm (the extinction coefficient was 4530 M^−1^cm^−1^) [45]. The reaction mixture contained 200 mmol L^−1^ potassium phosphate (pH 7.0), deionized water, and crude extract. The reaction was started by adding 40 μL β-isopropylmalate solution (40 mmol L^−1^ in deionized water). One unit of TA activity was defined as the activity necessary to form 1μmol of α-isopropylmaleate per min.

TA activity was determined at 30 °C by monitoring leucine formation by amination of α-ketoisocaproate using l-glutamate as an amino-group donor. The reaction mixture contained 100 mmol L^−1^ Tris-HCl (pH 9.0), 5 mmol L^−1^ sodium α-ketoisocaproate, 10 mmol L^−1^ sodium glutamate, 0.25 mmol L^−1^ pyridoxal-5′-phosphate, and crude extract [23]. The reaction was terminated by adding 21% perchloric acid. l-leucine formation was quantified by high-pressure liquid chromatography (HPLC) after neutralization with 5 mol L^−1^ KOH and centrifugation (8000× *g*, 10 min) at 4 °C. One unit of TA activity was defined as the activity necessary to form 1μmol of l-leucine per min.

LeuDH activity was measured at 30 °C by monitoring the oxidation of NADPH at 340 nm. The reaction mixture contained 100 mmol L^−1^ glycine-NaOH (pH 9.5), 10 mmol L^−1^ sodium α-ketoisocaproate, 0.2 mmol L^−1^ NADH, 200 mmol L^−1^ NH_4_Cl, and crude extract [23]. One unit of LeuDH activity was defined as the activity required to oxidize 1 μmol of NADH per min.

GDH activity was determined at 25 °C, by monitoring the oxidation of NADPH (or NADH) at 340 nm. The reaction mixture contained 55 mmol L^−1^ Tris-HCl (pH 7.5) with 2% glycerol, 10 mmol L^−1^ α-ketoglutarate, 10 mmol L^−1^ L NaCl, 100 mmol L^−1^ NH_4_Cl, 0.2 mmol L^−1^ NADPH (or NADH), and crude extract [23]. One unit of glutamate dehydrogenase activity was defined as the amount of enzyme that catalyzed the oxidation of 1 mmol NADPH (or NADH) per min.

### 4.3. Purification and Kinetic Analysis of AHAIR

Wild-type and mutant AHAIR were purified from BL21/pET28a-*ilvC* and BL21/pET28a-*ilvC*^TM^, and all purification steps were performed at 4 °C. Cultivated cells were disrupted by sonication and centrifuged to remove cell debris. Then, extract was purified by a protein purifier (AKTA Explorer 10, GE Healthcare, Uppsala, Sweden). Purified wild-type AHAIR and mutant AHAIR were used to perform enzyme activity for kinetic analysis. The decrease of NAD(P)H was measured at 340 nm. The α-acetolactate and NAD(P)H concentrations were from 0 to 20 mmol L^−1^ and 0 to 1 mmol L^−1^, respectively.

### 4.4. NADPH/NADP^+^ Assay

The cultivated cells were harvested by centrifugation (8000× *g*, 10 min) at 4 °C, and then cells were washed three times, to remove the residual extracellular metabolites, with ice-cold quenching solution (60% MeOH and 70 mmol L^−1^ HEPES). Intracellular NADPH and NADP^+^ were extracted and quantified using the Coenzyme II NADP(H) Content Assay kit (Solarbio, Beijing, China), respectively, following the manufacturer’s instructions. Then, the NADPH/NADP^+^ ratio was calculated.

### 4.5. Analytical Methods

Samples were taken every 4 h to determine some parameters. Cell growth was monitored by measuring the optical density of the culture at 562 nm (OD_562_), using a spectrophotometer (721N, shanghai, China), after diluting 0.2 mL of the sample with 5 mL 0.25 mol L^−1^ HCl, to dissolve the CaCO_3_ [46]. Glucose concentration was determined by SBA-40E immobilized enzyme biosensor (Biology Institute of Shandong Academy of Sciences, Jinan, China). Organic acid concentrations were determined by high-pressure liquid chromatography (HPLC) on an Agilent 1200 system (Agilent Technologies, Santa Clara, CA, USA) with UV detection (215 nm). Amino acid concentrations were determined by HPLC (Agilent Technologies, Santa Clara, CA, USA) with DAD detection (338 nm), after automatic precolumn derivatization with *ortho*-phthaldialdehyde [17].

## Figures and Tables

**Table 1:** Enzyme activities for the l-leucine biosynthesis by *C. glutamicum* strains.

Strains	Enzyme Activity (mU·mg_protein_^−1^)							
	IPMS	IPMI	AHAS	AHAIR	TA	LeuDH	GDH	RocG
XQ-9	262 ± 6	37 ± 5	143 ± 9	31 ± 8	17 ± 3	-	131 ± 8	-
ΔLtbR	289 ± 6	167 ± 8	139 ± 8	32 ± 5	19 ± 2		136 ± 5	
ΔLtbR/ABNCE	660 ± 13	153 ± 6	312 ± 14	74 ± 10	43 ± 5	-	134 ± 10	-
ΔLtbR-AHAIR^M^/ABNC^M^E	697 ± 23	151 ± 4	264 ± 10	63 ± 12	37 ± 4	-	135 ± 12	-
ΔLtbR-AHAIR^M^LeuDH/ABNC^M^LDH	528 ± 18	149 ± 5	196 ± 8	49 ± 9	-	446 ± 43	126 ± 5	-
ΔLtbR-AHAIR^M^LeuDHRocG/ABNC^M^LDH	489 ± 13	136 ± 4	185 ± 12	97 ± 11	-	414 ± 16	-	107 ± 8

All data represent values of three determinations of triplicate independent experiments with ±SEM.

**Table 2:** Kinetic parameters of the wild-type and mutant AHAIRs for NADH or NADPH ^a^.

AHAIR	NADH			NADPH		
	*Km* (μmol L^−1^)	*kcat* (s^−1^)	*kcat/Km* (s^−1^/mmol L^−1^)	*Km* (μmol L^−1^)	*kcat* (s^−1^)	*kcat/Km* (s^−1^/mmol L^−1^)
Wild-type	256 ± 13	0.93 ± 0.05	3.63	6.16 ± 1.34	0.6 ± 0.03	97.40
Mutant	276 ± 16	0.74 ± 0.03	2.68	67 ± 7	0.03 ± 0.0012	0.45

^a^ The α-acetolactate concentrations were 10 mmol L^−1^ and variable amounts of NAD(P)H were present. All data represent values of three determinations of triplicate independent experiments with ±SD.

**Table 3:** Bacterial strains and plasmids used in this study.

Strain or Plasmid	Relevant Characteristics	Source or Reference
Strains		
*E. coli*		
JM109	*recA1 end1 gyrA96 thi hsdR17 supE44 relA1* *Δ(lac-proAB)/F’(traD36 proAB^+^ lac^q^ lacZ* *Δ*M15*)*	Lab stock
BL21(DE3)	F- ompT gal dcm lon hsdS_B_ (r_B_- m_B_-) λ(DE3)	Lab stock
*Bacillus subtilis* 168	Wild type	ATCC
*C. glutamicum*		
Wild type	Wild type ATCC 13032, biotin auxotrophic	ATCC
XQ-9	l-leucine producing *C. glutamicum* strain created by random mutagenesis; resistant to α-thiazolealanine and α-aminobutyic acid; auxotrophic for l-isoleucine and l-methionine; can produce 14.12 g·L-^1^ l-leucine	Lab stock
ΔLtbR	*C. glutamicum* XQ-9 derivative with in-frame deletion of *ltbR*	This study
ΔLtbR/pECXK99E	*C. glutamicum* ΔLtbR harboring pECXK99E	This study
ΔLtbR/ABNCE	*C. glutamicum* ΔLtbR harboring pEC- ABNCE	This study
ΔLtbRAHAIR^M^	*C. glutamicum* ΔLtbR derivative with chromosomally integrated mutations into *ilvC*^M^ coding for amino acid exchanges S34G, L48E, and R49F	This study
ΔLtbRAHAIR^M^/ABNC^M^E	*C. glutamicum* ΔLtbRAHAIR^M^ harboring pEC-ABNC^M^E	This study
ΔLtbRAHAIR^M^LeuDH	*C. glutamicum* ΔLtbRAHAIR^M^ derivative with chromosomal integration of *leuDH* under control of the *tac* promoter integrated into the intergenic region between upstream and downstream of *ilvE*	This study
ΔLtbRAHAIR^M^LeuDH/ABNC^M^ELDH	*C. glutamicum* ΔLtbRAHAIR^M^LeuDH harboring pEC- ABNC^M^LDH	This study
ΔLtbRAHAIR^M^LeuDHRocG	*C. glutamicum* ΔLtbRAHAIR^M^LeuDH derivative with chromosomal integration of *rocG* under control of the *tac* promoter integrated into the intergenic region between upstream and downstream of *gdh*	This study
ΔLtbRAHAIR^M^LeuDHRocG/ABNC^M^LDH	*C. glutamicum* ΔLtbRAHAIR^M^LeuDHRocG harboring pEC- ABNC^M^LDH	This study
Plasmids		
pDXW-8	Kan^r^, *E. coli*-*C. glutamicum* shuttle vector for inducible gene expression (P*tac*, lacl,)	Lab stock
pET28a	Kan^r^, *E. coli* expression vector, PT7, pBR322orivector for inducible gene expression	Lab stock
pECXK99E	Kan^r^, *E. coli*-*C. glutamicum* shuttle vector for inducible gene expression	Lab stock
pK18*mobsacB*	Kan^r^, integration vector; *oriV_Ec_ oriT sacB*, allows for selection of double crossover *C. glutamicum*	Lab stock
pET28a-*ilvC*	Kan^r^, pET28a derivative containing gene *ilvC* (*C. glutamicum*)	
pET28a-*ilv*C^M^	Kan^r^, pET28a derivative with integrated mutations into *ilvC* coding for amino acid exchanges S34G, L48E, and R49F	
pEC-ABNCE	Kan^r^, pECXK99E derivative containing gene *leuA* (*C. glutamicum*), *ilvBNC* (*C. glutamicum*) and *ilvE* (*C. glutamicum*)	This study
pEC-ABNC^M^E	Kan^r^, pEC-ABNCE derivative integrated mutations into *ilvC* coding for amino acid exchanges S34G, L48E, and R49F	This study
pEC-ABNC^M^LDH	Kan^r^, pEC-ABNCLDH derivative with integrated mutations into *ilvC* coding for amino acid exchanges S34G, L48E, and R49F	This study
pDXW-8-*leuDH*	Kan^r^, pDXW-8 derivative containing gene *leuDH* (*Lysinibacillus sphaericus*)	This study
pDXW-8-*rocG*	Kan^r^, pDXW-8 derivative containing gene *rocG* (*Bacillus subtilis*)	This study
pK18*mobsacB*-Δ*ltbR*	Kan^r^, pK18*mobsacB* derivative for in-frame deletion of gene *ltbR*	This study
pK18*mobsacB*-*ilvC*^M^	Kan^r^, pK18*mobsacB* derivative with chromosomally integrated mutations into *ilvC* coding for amino acid exchanges S34G, L48E, and R49F	This study
pK18*mobsacB*-Δ*ilvE*::*leuDH*	Kan^r^, pK18*mobsacB*-Δ*ilvE* derivative containing *leuDH* gene from pDXW-8-*leuDH* under control of the *tac* promoter	This study
pK18*mobsacB*-Δ*gdh*::*rocG*	Kan^r^, pK18*mobsacB*-Δ*gdh* derivative containing *rocG* gene from pDXW-8-*rocG* under control of the *tac* promoter	This study

## Supplementary Materials

Supplementary materials can be found at https://www.mdpi.com/1422-0067/20/8/2020/s1.
