# Supplementary material for: Improvement of l-Leucine Production in Corynebacterium glutamicum by Altering the Redox Flux
Source: Int J Mol Sci. 2019 Apr 24;20(8):2020. doi: 10.3390/ijms20082020 (PMC6515235; doi:10.3390/ijms20082020)
Supplement: Supplementary file 1 [file ijms-20-02020-s001.pdf]

International Journal of *Molecular Sciences*

MDPI

Supplementary file

Improvement of L-Leucine Production in *Corynebacterium glutamicum* by Altering the Redox Flux

Ying-Yu Wang [1], Feng Zhang [1], Jian-Zhong Xu [1,2,*], Wei-Guo Zhang [1,*] and Xiu-Lai Chen [3] and Li-Ming Liu [3]

[1] The Key Laboratory of Industrial Biotechnology, Ministry of Education, School of Biotechnology, Jiangnan University, 1800# Lihu Road, WuXi 214122, China, wangyy0711@163.com (Y.-Y.W.); zhang20180606@sina.com (F.Z.)
[2] The Key Laboratory of Carbohydrate Chemistry and Biotechnology, Ministry of Education, School of Biotechnology, Jiangnan University, 1800# Lihu Road, WuXi 214122, China
[3] State key Laboratory of Food Science and Technology, Jiangnan University, Wuxi 214122, China; shirleyl1205@163.com (X.-L.C.), peidongchen13@163.com (L.-M.L.)
* Correspondence: zhangwg168@163.com (W.-G.Z.); xujianzhong@jiangnan.edu.cn(J.-Z.X.); Tel/Fax: +86-510-85329312(W.-G.Z.)

---

1. sequence

***leuA* sequence**

1 ATGCCAGTTA ACCGCTACAT GCCTTTCGAG GTTGAGGTAG AAGATATTTC TCTGCCGGAC
61 CGCACTTGGC CAGATAAAAA AATCACCGTT GCACCTCAGT GGTGTGCTGT TGACCTGCGT
121 GACGGCAACC AGGCTCTGAT TGATCCGATG TCTCCTGAGC GTAAGCGCCG CATGTTTGAG
181 CTGCTGGTTC AGATGGGATT CAAGGAAATC GAGGTCGGTT TCCCTTCAGC TTCCCAGACT
241 GATTTTGATT TCGTTCGTGA GATCATCGAA AAGGACATGA TCCCTGACGA TGTCACCATT
301 CAGGTTCTGG TTCAGGCTCG TGAGCACCTG ATTCGCCGTA CTTTTGAAGC TTGCGAAGGC
361 GCAAAAAACG TTATCGTGCA CTTCTACAAC TCAACCTCCA TCCTGCAGCG CAACGTGGTG
421 TTCCGCATGG ACAAGGTGCA GGTGAAGAAG CTGGCTACCG ATGCCGCTGA ACTGATCAAG
481 ACCGTCGCTC AGGATTACCC AGACACCAAC TGGCGCTGGC AGTACTCCCC TGAGTCCTTC
541 ACCGGCACTG AGGTTGAGTA CGCCAAGGAA GTTGTGGACG CAGTTGTTGA GGTCATGGAT
601 CCAACTCCTG AGAACCCAAT GATCATCGAC CTGCCTTCCA CCGTTAAGAT GATCACCCCT
661 AACGTTTACG CAGACTCCAT TGAATGGATG CACCGCAATC TAAACCGTCG TGATTCCATT
721 ATCCTGTCCC TGCACCCGCA CAATGACCGT GGCACCGGCG TTGGCGCGGC TGAGCTGGGC
781 TACATGGCTG GCGCTGACCG CATCGAAGGC TGCCTGTTCG GCAACGGCGA GCGCACCGGC
841 AACGTCTGCC TGGTCACCCT GGCACTGAAC ATGCTGACCC AGGGCGTTGA CCCTCAGCTG
901 GACTTCACCG ATATACGCCA GATCCGCAGC ACCGTTGAAT ACTGCAACCA GCTGCGCGTT
961 CCTGAGCGCC ACCCATACGG CGGCGACCTG GTCTTCACCG CTTTCTCCGG TTCCCACCAG
1021 GACGCTGTGA ACAAGGGTCT GGACGCCATG GCTGCCAAGG TTCAGCCAGG TGCTAGCTCC
1081 ACTGAAGTTT CTTGGGAGCA GCTGCGCGAC ACCGAATGGG AGGTTCCTTA CCTGCCTATC
1141 GATCCAAAGG ATGTCGGTCG CGACTACGAG GCTGTTATCC GCGTGAACTC CCAGTCCGGC
1201 AAGGGCGGCG TTGCTTACAT CATGAAGACC GATCACGGTC TGCAGATCCC TCGCTCCATG
1261 CAGGTTGAGT TCTCCACCGT TGTCCAGAAC GTCACCGACG CTGAGGGCGG CGAGGTCAAC
1321 TCCAAGGCAA TGTGGGATAT CTTCGCCACC GAGTACCTGG AGCGCACCGC ACCAGTTGAG
1381 CAGATCGCGC TGCGCGTCGA GAACGCTCAG ACCGAAAACG AGGATGCATC CATCACCGCC

1441 GAGCTCATCC ACAACGGCAA GGACGTCACC GTCGATGGCC ACGGCAACGG CCCACTGGCT
1501 GCTTACGCCA ACGCGCTGGA GAAGCTGGGC ATCGACGTTG AGATCCAGGA ATACAACCAG
1561 CACGCCCGCA CCTCGGACGA CGATGCAGAA GCAGCCGCCT ACGTGCTGGC TGAGGTCAAC
1621 GGCCGCAAGG TCTGGGGCGT CGGCATCGCT GGCTCCATCA CCTACGCTTC GCTGAAGGCA
1681 GTGACCTCCG CCGTAAACCG CGCGCTGGAC GTCAACCACG AGGCAGTCCT GGCTGGCGGC
1741 GTTTAA

***ilvB* sequence**

1 GTGAATGTGG CAGCTTCTCA ACAGCCCACT CCTGCCACGG TTGCAAGCCG TGGTCGATCC
61 GCCGCCCCTG AGCGGATGAC AGGTGCACAG GCAATTGTTC GATCGCTCGA GGAGCTTAAC
121 GCCGACATCG TGTTCGGTAT TCCTGGTGGT GCGGTGCTAC CGGTGTATGA CCCGCTCTAT
181 TCCTCCACAA AGGTGCGCCA CGTCTTAGTG CGCCACGAGC AGGGCGCAGG CCACGCAGCA
241 ACCGGCTACG CGCAGGTTAC TGGACGCGTT GGCGTCTGCA TTGCAACCTC TGGCCCAGGC
301 GCAACCAACT TGGTTACCCC AATCGCTGAT GCAAACTTGG ACTCCGTTCC CATGGTTGCC
361 ATCACCGGCC AGGTCGGAAG TAGCCTGCTG GGTACCGATG CTTTCCAGGA AGCCGATATC
421 CGCGGCATCA CCATGCCAGT GACCAAGCAC AACTTCATGG TCACCAACCC CAACGACATT
481 CCACAGGCAT TGGCTGAGGC ATCCCACCTC GCGATTACTG GTCGCCCTGG TCCTGTTCTA
541 GTGGATATCC CCAAGGATGT TCAGAACGCT GAATTGGATT TCGTCTGGCC ACCAAAGATC
601 GACCTGCCAG GCTACCGCCC AGTTTCAACA CCGCATGCTC GACAGATTGA GCAGGCTGTC
661 AAACTGATCG GTGAGTCTAA GAAGCCTGTC CTTTACGTTG GCAGCGGCGT TATCAAGGCT
721 GATGCCCACG AAGAGCTTCG TGCGTTCGTT GAGCACACCG GCATTCCAGT TGTCACCACA
781 TTGATGGCGC TGGGAACCTT CCCAGAGTCC CACGAGCTGC ACATGGGTAT GCCAGGCATG
841 CATGGCACTG TGTCCGCTGT TGGTGCACTG CAGCGCAGCG ACCTGCTGAT TGCTATCGGC
901 TCCCGCTTTG ATGACCGCGT CACCGGTGAC GTTGACACTT TCGCACCTGA TGCCAAGATC
961 ATTCACGCCG ACATTGATCC TGCCGAAATC GGCAAGATCA AGCAGGTTGA GGTTCCAATC
1021 GTGGGCGATG CCCGCGAGGT TCTTGCTCGT CTGCTCGAAA CCACCAAGGC AAGCAAGGCA
1081 GAGTCTGAGG ACACCTCCGA GTGGGTTGAC TACCTCAAGG GCCTCAAGGC ACGTTTCCCA
1141 CGTGGCTACG ACGAGCAGCC AGGCGATCTG CTGGCACCAC AGTTTGTCAT TGAAACCCTG
1201 TCCAAGGAAG TTGGCCCCGA CGCAATTTAC TGCGCCGGCG TTGGCCAGCA CCAGATGTGG
1261 GCAGCTCAGT TCGTTGACTT CGAAAAGCCA CGCACCTGGC TCAACTCCGG TGGACTGGGC
1321 ACCATGGGCT ACGCAGTTCC TGCGGCTCTT GGAGCAAAGG CTGGCGCACC TGACAAGGAA
1381 GTCTGGGCTA TCGACGGCGA CGGCTGTTTC CAGATGACCA ACCAGGAACT CACCACCGCC
1441 GCAGTTGAAG GTTTCCCCAT TAAGATCGCA CTAATCAACA ACGGAAACCT GGGTATGGTT
1501 CGCCAATGGC AGACCCTATT CTATGAAGGA CGGTACTCAA ATACTAAACT TCGTAACCAG
1561 GGCGAGTACA TGCCCGACTT TGTTACCCTT CCTGAGGGAC TTGGCTGTGT TGCCATCCGC
1621 GTCACCAAAG CGGAGGAAGT ACTGCCAGCC ATCCAAAAGG CACGAGAGAT CAACGACCGC
1681 CCAGTAGTCA TCGACTTCAT CGTCGGTGAA GACGCACAGG TATGGCCAAT GGTGTCTGCT
1741 GGATCATCCA ACTCCGATAT CCAGTACGCA CTCGGATTGC GCCCATTCTT TGATGGTGAT
1801 GAATCTGCAG CAGAAGATCC TGCCGACATT CACGAAGCCG TCAGCGACAT TGATGCCGCC
1861 GTTGAATCGA CCGAGGCATA A

***ilvN* sequence**

1 ATGGCTAATT CTGACGTCAC CCGCCACATC CTGTCCGTAC TCGTTCAGGA CGTAGACGGA
61 ATCATTTCCC GCGTATCAGG TATGTTCACC CGACGCGCAT TCAACCTCGT GTCCCTCGTG
121 TCTGCAAAGA CCGAAACACT CGGCATCAAC CGCATCACGG TTGTTGTCGA CGCCGACGAG

181 CTCAACATTG AGCAGATCAC CAAGCAGCTC AACAAGCTGA TCCCCGTGCT CAAAGTCGTG
241 CGACTTGATG AAGAAACCAC TATCGCCCGC GCAATCATGC TGGTTAAGGT TTCTGCGGAC
301 AGCACCAACC GTCCGCAGAT CGTCGACGCC GCGAACATCT TCCGCGCCCG AGTCGTCGAC
361 GTGGCTCCAG ACTCTGTGGT TATTGAATCC ACAGGCACCC CAGGCAAGCT CCGCGCACTG
421 CTTGACGTGA TGGAACCATT CGGAATCCGC GAACTGATCC AATCCGGACA GATTGCACTC
481 AACCGCGGTC CGAAGACCAT GGCTCCGGCC AAGATCTAA

***ilvC* sequence**

1 ATGGCTATTG AACTGCTTTA TGATGCTGAC GCTGACCTCT CCTTGATCCA GGGCCGCAAG
61 GTTGCCATCG TTGGCTACGG CTCCCAGGGC CACGCACACT CCCAGAACCT CCGCGATTCT
121 GGCGTTGAGG TTGTCATTGG TCTGCGCGAG GGCTCCAAGT CCGCAGAGAA GGCAAAGGAA
181 GCAGGCTTCG AGGTCAAGAC CACCGCTGAG GCTGCAGCTT GGGCTGACGT CATCATGCTC
241 CTGGCTCCAG ACACCTCCCA GGCAGAAATC TTCACCAACG ACATCGAGCC AAACCTGAAC
301 GCAGGCGACG CACTGCTGTT CGGCCACGGC CTGAACATTC ACTTCGACCT GATCAAGCCA
361 GCTGACGACA TCATCGTTGG CATGGTTGCG CCAAAGGGCC CAGGCCACTT GGTTCGCCGT
421 CAGTTCGTTG ATGGCAAGGG TGTTCCTTGC CTCATCGCAG TCGACCAGGA CCCAACCGGA
481 ACCGCACAGG CTCTGACCCT GTCCTACGCA GCAGCAATCG GTGGCGCACG CGCAGGCGTT
541 ATCCCAACCA CCTTCGAAGC TGAGACCGTC ACCGACCTCT TCGGCGAGCA GGCTGTCCTC
601 TGCGGTGGCA CCGAGGAACT GGTCAAGGTT GGCTTCGAGG TTCTCACCGA AGCTGGCTAC
661 GAGCCAGAGA TGGCATACTT CGAGGTTCTT CACGAGCTCA AGCTCATCGT TGACCTCATG
721 TTCGAAGGTG GCATCAGCAA CATGAACTAC TCTGTTTCTG ACACCGCTGA GTTCGGTGGC
781 TACCTCTCCG GCCCACGCGT CATCGATGCA GACACCAAGT CCCGCATGAA GGACATCCTG
841 ACCGATATCC AGGACGGCAC CTTCACCAAG CGCCTCATCG CAAACGTTGA GAACGGCAAC
901 GCCGAGCTTG AGGGCCTTCG CGCTTCCTAC AACAACCACC CAATCGAGGA GACCGGCGCT
961 AAGCTCCGCG ACCTCATGAG CTGGGTCAAG GTTGACGCTC GCGCAGAAAC CGCTTAA

***ilvE* sequence**

1 GTGTATCTGT CAGGTAGCAG GTGTACCTTG AAATCCATGA CGTCATTAGA GTTCACAGTA
61 ACCCGTACCG AAAATCCGAC GTCACCCGAT CGTCTGAAGG AAATTCTTGC CGCACCGAAG
121 TTCGGTAAGT TCTTCACCGA CCACATGGTG ACCATTGACT GGAACGAGTC GGAAGGCTGG
181 CACAACGCCC AATTAGTGCC ATACGCGCCG ATTCCTATGG ATCCTGCCAC CACCGTATTC
241 CACTACGGAC AGGCAATTTT TGAGGGAATT AAGGCCTACC GCCATTCGGA CGAAACCATC
301 AAGACTTTCC GTCCTGATGA AAACGCCGAG CGTATGCAGC GTTCAGCAGC TCGAATGGCA
361 ATGCCACAGT TGCCAACCGA GGACTTTATT AAAGCACTTG AACTGCTGGT AGACGCGGAT
421 CAGGATTGGG TTCCTGAGTA CGGCGGGGAA GCGTCCCTCT ACCTGCGCCC ATTCATGATC
481 TCCACCGAAA TTGGCTTGGG TGTCAGCCCA GCTGATGCCT ACAAGTTCCT GGTCATCGCA
541 TCCCCAGTCG GCGCTTACTT CACCGGTGGA ATTAAGCCTG TTTCCGTCTG GCTGAGCGAA
601 GATTACGTCC GCGCTGCACC CGGCGGAACT GGTGACGCCA AATTTGCTGG CAACTACGCG
661 GCTTCTTTGC TTGCCCAGTC CCAGGCTGCG GAAAAGGGCT GTGACCAGGT CGTATGGTTG
721 GATGCCATCG AGCACAAGTA CATCGAAGAA ATGGGTGGCA TGAACCTTGG GTTCATCTAC
781 CGCAACGGCG ACCACGTCAA GCTAGTCACC CCTGAACTTT CCGGCTCACT ACTTCCAGGC
841 ATCACCCGCA AGTCACTTCT ACAAGTAGCA CGCGACTTGG GCTACGAAGT AGAAGAGCGA
901 AAGATCACCA CCACCGAGTG GGAAGAAGAC GCAAAGTCTG GCGCCATGAC TGAGGCATTT
961 GCTTGCGGTA CTGCAGCTGT TATCACCCCT GTTGGCACCG TGAAATCAGC TCACGGCACC
1021 TTCGAAGTGA ACAACAATGA AGTCGGAGAA ATCACGATGA AGCTTCGTGA AACCCTCACC

1081 GGAATTCAGC AAGGAAACGT TGAAGACCAA AACGGATGGC TTTACCCACT GGTTGGCTAA

Table S1. The NADPH concentration of *C. glutamicum* strains

Strains	NADPH (μmol·g^{-1})
XQ-9	2.78±0.16
△LtbR/ABNCE	3.48±0.21
△LtbR-AHAIR^{M}/ABNC^{M}E	3.92±0.33
△LtbR-AHAIR^{M}LeuDH/ABNC^{M}LDH	5.34±0.26
△LtbR-AHAIR^{M}LeuDHRocG/ABNC^{M}LDH	5.61±0.17

The data are averages from triplicate experiments.

Table S2. Oligonucleotides used in this study

oligonucleotides	Sequence(5′-3′)[a]	Overhung restriction sites[b]
leuA-F	CCGGAATTCGAAAGGAGATATACCATGCCAGTTAACCGCT	*Eco*RI
leuA-R	CGGGGTACCAGGCGTCGTAAAGCT	*Kpn*I
ilvBNC-F	TCCCCCGGGGAAAGGAGATATACCGTGAATGTGGCAGCTTCTC	*Sma*I
ilvBNC-R	GCTCTAGATTAAGCGGTTTCTGCG	*Xba*I
ilvE-F	GCTCTAGAGAAAGGAGATATACCGTGTATCTGTCAGGTAGCAGGTGT	*Xba*I
ilvE-R	GCTCTAGATTAGCCAACCAGTGGGTAAAGCCAT	*Xba*I
rocG -F	CGAGCTCGAAAGGAGATATACCATGTCAGCAAAGCAAG	*Sac*I
rocG -R	CCCAAGCTTTTAGACCCATCCGCGG	*HindIII*
P_{tac}- *rocG* -F	GCTCTAGACATCATAACGGTTCTGG	*Xba*I
P_{tac}- *rocG* -R	ACGCGTCGACACCATCGGCGCTACGG	*Sal*I
P_{tac}-leuDH-F	GCTCTAGAACCATCGGCGCTACGG	*Xba*I
P_{tac}-leuDH-R	GCTCTAGACATCATAACGGTTCTGG	*Xba*I
ilvC-F	CCGGAATTCATGGCTATTGAACTGCT	*Eco*RI
ilvC-R	CCCAAGCTTTTAAGCGGTTTCTGCG	*Hind*III
*ilv*CM1-F	GGCCACGCACAC***GGC***CAGAACCTCCGCGATTCTG	
*ilv*CM1-R	CAGAATCGCGGAGGTTCTG***GCC***GTGTGCGTGGCC	
*ilv*CM2-F	GAGGTTGTCATTGGT***GAGTTC***GAGGGCTCCAAGTCC	
*ilv*CM2-R	GGACTTGGAGCCCTC***GAACTC***ACCAATGACAACCTC	
ltbR-U-F	CCCAAGCTTTCACAGTTGTCGCGCAG	*Hind*III
ltbR-U-R	*AAGCTCACCTTGAAT*TTAGCATTTCACCC	
ltbR-D-F	*ATTCAAGGTGAGCTT*TTCGACGCAC	
ltbR-D-R	CGCGGATCCGATCGGATTCCTGGCT	*Bam*HI
ilvE-U-F	TCCCCCGGGCAAGCCTAGCCATTCCTC	*Sma*I
ilvE-U-R	GCTCTAGACGTCTACCAGCAGTTCAAG	*Xba*I
ilvE-D-F	GCTCTAGATGGGATACGAAGTAGAAGAGC	*Xba*I
ilvE-D-R	ACGCGTCGACTTTCCAACCGTCAGCTG	*Sal*I
gdh-U-F	TCCCCCGGGACCAGCCTAAATGCCCG	*Sma*I
gdh-U-R	GCTCTAGATGACGTGGACCTGGCC	*Xba*I
gdh-D-F	ACGCGTCGACTCGGACCAGGCAAGG	*Sal*I
gdh-D-R	GCCTGCAGTTAGATGCTGACGCCC	*Pst*I

[a] mutation sites in oligonucleotides are bolded, complementary sequences in oligonucleotides are italicized.
[b] each overhung restriction sites used for cloning procedure is underlined.

2. Plasmid Constructions

According to standard protocols, routine methods of molecular cloning like PCR, DNA restriction, and ligation were carried out [1].

Vector pK18*mobsacB*-△*ltbR* was constructed via an overlap-extension PCR—the region upstream (approximately 500 bp) of the *ltbR*, as well as the downstream region (approximately 690 bp) of *ltbR*, were amplified via oligonucleotide pairs *ltbR*-U-F/ *ltbR*-U-R and *ltbR*-D-F/ *ltbR*-D-R, respectively, from genomic DNA of *C. glutamicum* XQ-9. The amplified DNA fragments were fused in an overlap-extension PCR, resulting in a 1190 bp PCR product that was amplified with *ltbR*-U-F and *ltbR*-D-R. This fragment was digested with the restriction enzymes *Hin*dIII and *Bam*HI and ligated into the equally digested vector pK18*mobsacB*.

The plasmid pK18*mobsacB*-△*ilvE::leuDH* was constructed as follows—the region upstream (approximately 490 bp) of *ilvE*, as well as the downstream region (approximately 530 bp) of *ilvE*, were amplified via oligonucleotide pairs *ilvE*-U-F/*ilvE*-U-R and *ilvE*-D-F/*ilvE*-D-R, respectively, from genomic DNA of *C. glutamicum* XQ-9. The upstream fragment was digested with the restriction enzymes *Sma*I and *Xba*I and ligated into the equally digested vector pK18*mobsacB*. Then, the downstream fragment was digested with the restriction enzymes *Xba*I and *Sal*I and ligated into the equally digested vector pK18*mobsacB*-△*ilvE*-U. The *leuDH* gene (1095 bp) supplemented with an SD sequence (GAAAGGAGATATACC) before ATG was synthesized by General Biosystems Co. Ltd (Anhui, China) using pUC-57 as vector. Promoter and terminator elements can be used from pDXW-8. Thus, *leuDH* gene was digested with the restriction enzymes *Eco*RI and *Kpn*I and ligated into the vector pDXW-8 that had been digested with the same enzymes. The P_{tac}-*leuDH-rrnBT1T2* fragment was amplified via oligonucleotide pairs P_{tac}-*leuDH*-F/P_{tac}-*leuDH*-R from plasmid pDXW-8-*leuDH*. The fragment was digested with the restriction enzyme *Xba*I and ligated into the equally digested vector pK18*mobsacB*-△*ilvE*.

For construction of pEC-*leuAilvBNCE*, pEC-*leuAilvBNC*^{M}*E* and pEC-*leuAilvBNC*^{M}*leuDH*, the *leuA*, *ilvBNC*, and *ilvE* genes were amplified via oligonucleotides *leuA*-F/*leuA*-R, *ilvBNC*-F/*ilvBNC*-R, and *ilvE*-F/*ilvE*-R, respectively, from genomic DNA of *C. glutamicum* XQ-9. The *leuA* fragment was digested with the restriction enzymes *Eco*RI and *Kpn*I and ligated into the vector pECXK99E that had been digested with the same enzymes. The *ilvBNC* fragment was digested with the restriction enzymes *Sma*I and *Xba*I and ligated into the equally digested vector pEC-*leuA*. The *ilvE* fragment was digested with the restriction enzyme *Xba*I and ligated into the equally digested vector pEC-*leuAilvBNC*. Due to the single-restricted ligation, the correct pEC-*leuAilvBNCE* plasmid was verified by DNA sequencing. Site-directed mutagenesis was performed using the Mut ExpressR II Fast Mutagenesis Kit V2 (Vazyme, Nanjing, China), following the manufacturer's instructions. Using pEC-*leuAilvBNCE* as a template, PCR products were orderly amplified via oligonucleotides *ilv*CM1-F/ *ilv*CM1-R, and *ilv*CM2-F/ *ilv*CM2-R. Subsequently, methylated templates of the PCR

products were digested with *Dpn*I, and the resultant products were performed by recombinase Exnase II in vitro. The product could be amplified in vivo by transforming *E. coli*. The correctness of the plasmid resulting from site-directed mutagenesis was verified by DNA sequencing. For construction of pEC-*leuAilvBNC*^{M}*leuDH*, P_{tac}-*leuDH-rrnBT1T2* fragment was amplified via the oligonucleotide pair P_{tac}-*leuDH*-F/P_{tac}-*leuDH*-R from plasmid pDXW-8-*leuDH*. The fragment was digested with the restriction enzyme *Xba*I and ligated into the equally digested vector pEC-*leuAilvBNC*. The correctness of the plasmid resulting from site-directed mutagenesis was verified by DNA sequencing.

To obtain the vectors pET28a-*ilv*C, pET28a-*ilv*C^{M}, and pk18*mobsacB*-*ilv*C^{M}, a 1117 bp PCR fragment was generated from the genomic DNA of *C. glutamicum* wild type. It covered the whole sequence of *ilvC*. The fragment was cut with the restriction enzymes *Eco*RI and *Hin*dIII and ligated into the vectors pET28a and pk18*mobsacB* that had been digested with the same enzymes, respectively. Site-directed mutagenesis was performed using the Mut ExpressR II Fast Mutagenesis Kit V2 (Vazyme, Nanjing, China), following the manufacturer's instructions. Using pET28a-*ilv*C and pk18*mobsacB*-△*ilvC* as templates, PCR products was orderly amplified via oligonucleotides *ilv*CM1-F/ *ilv*CM1-R and *ilv*CM2-F/ *ilv*CM2-R. Subsequently, methylated templates of PCR products were digested with *Dpn*I, and the resultant products were performed by recombinase Exnase II in vitro. The product could be amplified in vivo by transforming *E. coli*. The correctness of the plasmid resulting from site-directed mutagenesis was verified by DNA sequencing.

For construction of vector pK18*mobsacB*-△*gdh::rocG*, the step was similar—amplifying the upstream and downstream region for *gdh* via oligonucleotides *ilvE*-U-F/*ilvE*-U-R and *ilvE*-D-F/*ilvE*-D-R, respectively, from the genomic DNA of *C. glutamicum* XQ-9. The upstream fragment was digested with the restriction enzymes *Sma*I and *Xba*I and ligated into the equally digested vector pK18*mobsacB*. Then, the downstream fragment was digested with the restriction enzymes *Sal* I and *Pst*I and ligated into the equally digested vector pK18*mobsacB*-△*gdh*-U. The *rocG* gene was amplified via the oligonucleotide pair *rocG*-F/*rocG*-R from genomic DNA of *Bacillus subtilis* 168. The *rocG* gene was digested with the restriction enzymes *Sac*I and *Hin*dIII and ligated into the vector pDXW-8 that had been digested with the same enzymes. The P_{tac}-*rocG-rrnBT1T2* fragment was amplified via oligonucleotide pair P_{tac}-*rocG*-F/P_{tac}-*rocG*-R from plasmid pDXW-8-*rocG*. The fragment was digested with the restriction enzymes *Sal*I and *Xba*I and ligated into the equally digested vector pK18*mobsacB*-△*gdh*.

Reference

1. Sambrook, J.; David, W. *Molecular Cloning: A Laboratory Manual*, 3nd ed.; Cold Spring Harbor Laboratory Press: New York, NY, USA, 2001; ISBN-10 0879695773, ISBN-13 978-0879695774.

© 2019 by the authors. Submitted for possible open access publication under the terms and conditions of the Creative Commons Attribution (CC BY) license (http://creativecommons.org/licenses/by/4.0/).
